# Buprenorphine initiation from fentanyl using low-dose intramuscular ketamine: a pilot study

**DOI:** 10.1186/s13722-026-00649-3

**Published:** 2026-02-11

**Authors:** J. Luke Engeriser, Thomas Hutch, Crystal L. Smith, Zach Orme, Evan Chavers, Lucinda A. Grande

**Affiliations:** 1 AltaPointe Health, 5750-A Southland Drive, Mobile, AL 36693 USA; 2 University of South Alabama Frederick P. Whiddon College of Medicine, 1015 Montlimar Drive, Suite A-210, Mobile, AL 36609 USA; 3 We Care Daily Clinics, Seattle, WA USA; 4 Department of Family Medicine, University of Washington School of Medicine, 1959 N.E. Pacific St., Box 356390, Seattle, WA 98195 USA; 5 Washington State University, 412 E Spokane Falls Blvd., Spokane, WA 99202 USA; 6 Memorial Hospital, 1900 State Street, Chester, IL 62233 USA; 7 Pioneer Family Practice, 5130 Corporate Ctr Ct SE, Lacey, WA 98503 USA

**Keywords:** Fentanyl, Buprenorphine, Ketamine, Precipitated withdrawal, Opioid use disorder

## Abstract

**Background:**

Buprenorphine is an effective medication for treating opioid use disorder but is underutilized partly due to patient apprehensiveness of the severe withdrawal symptoms it can induce when started, particularly by fentanyl-dependent individuals. An emerging facilitator to buprenorphine initiation is ketamine, a dissociative anesthetic shown to reverse opioid withdrawal symptoms in case reports and small series. A 24-hour behavioral health crisis center implemented a quality improvement protocol to address difficulties transitioning patients from fentanyl to buprenorphine using a high-dose buprenorphine initiation strategy premedicated with a low, sub-dissociative dose of intramuscular ketamine.

**Methods:**

Crisis center personnel injected ketamine 10 mg intramuscularly at a cost of $0.44 per patient and 30 minutes later administered buprenorphine 8 mg sublingually to patients who self-reported recent use of fentanyl and were in at least moderate withdrawal. They assessed Clinical Opiate Withdrawal Scale (COWS) scores at baseline, 30 minutes after ketamine, and 30 minutes after buprenorphine. They compared length of stay (LOS) between patients initiating buprenorphine before and after implementing the protocol. They recorded pharmacy dispensing of buprenorphine prescriptions and follow-up visits within 30 days.

**Results:**

In 50 patients treated over 13 months, average COWS score dropped from 13.6 (range 8–21, SD 2.9) at baseline to 6.2 (range 0–17, SD 3.6) 30 minutes after ketamine and 4.1 (range 0–18, SD 4.0) 30 minutes after buprenorphine. Both decreases were statistically significant (*p* < 0.001) with very large effect sizes. Of the 50 patients, 36 (72%) experienced a decrease in COWS score including 27/50 (54%) with COWS score 0–3 by 30 minutes after buprenorphine. After protocol implementation, median LOS decreased from 66.0 hours (range 20–135) in the pre-protocol comparison sample to 7.0 hours (range 2–148) in the post-protocol sample. Patients reported no adverse effects of ketamine.

**Conclusions:**

In this observational study, sub-dissociative dose intramuscular ketamine was a well-tolerated and inexpensive premedication for a high-dose buprenorphine initiation strategy. Most patients had rapid reduction in signs of fentanyl withdrawal after ketamine, and half had nearly complete resolution following buprenorphine. Ketamine may be a useful adjunct to increase buprenorphine uptake and reduce overdose deaths. Prospective research is warranted.

## Key Findings

A) Average COWS scores at baseline, 30 minutes after ketamine, and 30 minutes after buprenorphine were 13.6, 6.2, and 4.1 respectively.

B) 30 minutes after buprenorphine (1 hour after starting the protocol), 49/50 patients (98%) had a lower COWS score, and 54% had COWS score 0–3 (minimal to no withdrawal). 3 patients required overnight stay due to inadequate control of withdrawal.

C) Median length of stay (in hours) at the facility decreased from 66.0 to 7.0 after starting the protocol.

## Background

Buprenorphine is an effective medication for treatment of opioid use disorder (OUD), reducing mortality risk by as much as 70% [1], while producing individual and societal benefits such as reductions in illicit opioid use [2] and healthcare utilization [3, 4]. However, it is underutilized in part due to patient apprehensiveness of the treatment initiation process [5]. In individuals with physiologic opioid dependence, starting buprenorphine can result in severe acute withdrawal symptoms, known as buprenorphine-precipitated opioid withdrawal (BPOW) [6, 7], a distressing condition traditionally avoided by a prolonged delay incurring spontaneous opioid withdrawal symptoms [8]. BPOW is thought to occur because of buprenorphine’s pharmacological trait as a partial mu-opioid receptor agonist with high receptor affinity [9]. It displaces full opioid agonists from the mu-opioid receptor, producing an abrupt drop in opioid effects which triggers a physiological withdrawal syndrome.

While prescription analgesics and heroin were dominant in the illicit opioid market in the U.S., a 12 hour interval after last opioid use was often adequate to avoid BPOW [8]. However, as illicitly-manufactured fentanyl (hereafter referred to as “fentanyl”) grew to dominance in the U.S. from 2013 to 2022 [10], many patients reported severe symptoms of BPOW occurring even 48 hours after last use [6]. Spontaneous withdrawal symptoms, while largely resolved by 24 hours after abstinence from non-fentanyl opioids, can be severe even after 5 days of abstinence from fentanyl [11].

Fentanyl’s pharmacological traits, including its high mu-opioid receptor binding affinity and high potency, are thought to be responsible for the heightened risk and severity of BPOW [12]. Its lipophilicity contributes to increased distribution into adipose tissue and prolonged release into the bloodstream and results in the prolonged BPOW vulnerability [12]. Reports of BPOW during the fentanyl era have contributed to patient and provider hesitancy to initiate buprenorphine treatment [13].

New initiation strategies have been explored to avoid BPOW and/or to manage spontaneous withdrawal symptoms during the prolonged period of vulnerability [14]. The strategies include low-dose (“microdose”) initiation [15], high-dose (“macrodose”) initiation [16], early initiation of long-acting injectable (LAI) buprenorphine [17, 18] and rapid buprenorphine rescue after naloxone-induced withdrawal [19, 20]. Since none of these methods has achieved reliable success in avoiding BPOW, shared decision-making between the clinician and patient has become increasingly important to improve patient satisfaction [21].

An emerging facilitator to buprenorphine initiation is the use of ketamine. Approved by the Food and Drug Administration (FDA) in 1970 as a dissociative anesthetic agent, intravenous ketamine has entered mainstream use at a sub-anesthetic dose for a variety of off-label applications, most prominently rapid relief from treatment-resistant depression [22] and alleviation of both acute pain [23] and chronic pain [24]. Recent literature shows efficacy of ketamine or its bioactive metabolites in treating different phases of substance use disorders. Early clinical studies demonstrate the effectiveness of ketamine in treatment of alcohol, cocaine, opioid and cannabis use disorders [25]. In OUD, studies demonstrate that ketamine can reduce cravings, opioid use, and precipitated withdrawal symptoms [26]. One comprehensive study in a mouse model [27] demonstrated the prevention efficacy of (2 R,6 R)-hydroxynorketamine (HNK), a ketamine metabolite currently in Phase II clinical trials at every stage of OUD including addiction, withdrawal, and protracted abstinence - sometimes referred to as post-acute withdrawal syndrome (PAWS) [28]. Specifically, (2 R,6 R)-HNK prevented behavior changes seen in the mouse model of naloxone-precipitated opioid withdrawal. These effects were accompanied by restoration of synaptic plasticity markers, possibly through NMDA receptor-mediated and high-frequency electroencephalographic oscillation mechanisms.

Although the ketamine molecule interacts with many receptor types, its primary pharmacological mechanism of action is thought to be its antagonist effect at the N-methyl-D-aspartate-type glutamate receptor (NMDAr) [29, 30]. Blocking the NMDAr has been shown in animal models to rapidly reverse the central nervous system adaptations that mediate opioid dependence, tolerance and withdrawal, and to enhance opioid signaling [31]. In addition, ketamine rapidly reverses opioid tolerance at the receptor level by resensitizing mu-opioid receptors previously desensitized by prolonged exposure to opioids [32–34]. By increasing both the concentration and efficacy of endogenous opioids, it can be viewed as functioning as a proxy mu-opioid agonist [34–36].

In a recent inpatient study, adults with both OUD and MDD were randomized to receive intravenous ketamine or high dose buprenorphine and then compared on measures of anxiety and opioid cravings 2 hours, 24 hours and 7 days later. Individuals in the ketamine group reported a rapid and substantial reduction in anxiety symptom severity within hours of administration, accompanied by a pronounced decline in opioid craving intensity [37]. In another randomized controlled trial, an intravenous infusion of ketamine markedly blunted objective signs of precipitated opioid withdrawal over three hours while opioid-dependent subjects underwent a naloxone challenge under general anesthesia to facilitate discontinuation of opioids [38].

Recent case reports have demonstrated the use of ketamine to treat established BPOW using an intravenous infusion at a sub-anesthetic dose over several hours in a hospital emergency department [39], an inpatient ward [40] and a pain clinic [41]. A pilot study demonstrated the feasibility of lower, sub-dissociative doses of sublingual (SL) ketamine over several days to relieve spontaneous opioid withdrawal symptoms and prevent BPOW during buprenorphine initiation in the ambulatory setting [42].

Patients sometimes undergo buprenorphine initiation in non-hospital settings such as residential treatment programs and high-acuity crisis centers [43], but a role for ketamine in buprenorphine initiation has not yet been explored in these settings which are more cost-effective for withdrawal management than emergency departments or inpatient wards. However, they often lack the ready accessibility of specialized medical supplies and personnel required to manage the undesirable risks of a ketamine infusion at an anesthetic or sub-anesthetic dissociative dose, including: 1) cardiovascular complications from elevated blood pressure and heart rate [44], and 2) psychiatric side-effects that could result in psychological distress and behavioral challenges [45].

The low SL dose of ketamine used in the outpatient pilot study avoided a ketamine-induced dissociative state [42]. However, use of the SL mode of administration introduces logistical challenges: commercially available liquid ketamine is bitter tasting, but the more palatable compounded ketamine troches and syrup are difficult to obtain quickly.

The high-dose buprenorphine initiation strategy avoids an extended time-course, which has two advantages: 1) it ensures administration of a therapeutic dose, avoiding the risk of early patient drop-out, and 2) it reduces the number of expensive days in an inpatient setting. In the emergency department setting, the high-dose strategy has been shown to avoid BPOW in most patients [16, 46–48]. However, the favorable results have not yet been duplicated in the outpatient setting, where the range of strategies available for managing persistent or worsening withdrawal symptoms may be more limited.

An optimal strategy for ketamine-assisted buprenorphine initiation (KABI) would therefore: 1) avoid the undesirable risks of dissociative dose ketamine, 2) use commercially available liquid ketamine, and 3) use a high-dose buprenorphine initiation strategy.

Intramuscular (IM) injection of sub-dissociative dose ketamine has the potential to accomplish all of these goals. It employs commercially available liquid ketamine, which is practical in any medical setting. In addition, it produces a rapid increase in plasma concentration that peaks within 5–30 minutes after injection, then falls to about 50% of its peak level by about an hour, with a half-life of 2–3 hours [30, 49]. This time course closely matches the time course of BPOW symptoms which peak within 60 minutes after buprenorphine exposure in 90% of people [50]. IM ketamine could therefore be an excellent premedication for a high-dose buprenorphine initiation.

The present report addresses critical gaps in the empirical literature on the utility of ketamine in buprenorphine initiation 1) as premedication for a high-dose initiation strategy, and 2) use of the IM mode of administration. It describes a quality improvement project designed to evaluate the feasibility, dosing, safety, and effectiveness of a sub-dissociative dose of IM ketamine premedication for reducing BPOW severity during a high-dose buprenorphine initiation at a 24-hour behavioral health crisis center. By systematically capturing clinically observed withdrawal symptoms and initiation outcomes, this project aimed to inform best practices and support the development of evidence-based initiation protocols tailored to the challenges of the fentanyl era.

## Methods

### Setting

Program leadership initiated a quality improvement pilot project at the AltaPointe Health 24-hour behavioral health crisis center (BHCC) in Mobile, Alabama to address difficulties in transitioning patients from fentanyl to buprenorphine. The BHCC is a 22-bed facility that opened in May 2021 as a medically monitored, short-term residential service offering 23-hour observation beds and a crisis stabilization unit for individuals experiencing a psychiatric crisis. Designed to provide round-the-clock observation, medication, therapy, and psychiatric care, the BHCC employs psychiatrists, psychiatric nurses, and other mental healthcare workers. The BHCC serves both urban and rural underserved communities in Mobile and the surrounding seven-county region.

Admissions to the BHCC come from a variety of referral sources: emergency departments, primary care and behavioral health outpatient clinics, law enforcement, direct patient calls to the Access to Care department, and walk-ins. The BHCC provides free of charge care for uninsured patients, and all admissions are voluntary. Individuals can stay in the BHCC for up to 8 days, and if continued care is required beyond this length of time, arrangements are made for transfer to a longer-stay facility. At the time of this project, medical providers were not available on-site after-hours, but there was always a physician available on-call. In addition to treating psychiatric emergencies such as suicidality, acute psychosis, and major mood episodes, the BHCC also treats individuals experiencing withdrawal from alcohol, opioids, and other substances. The BHCC offers initiation of buprenorphine for individuals using opioids. Following initiation, patients are offered referral to the AltaPointe substance use disorders clinic which provides comprehensive care including medications for OUD for individuals in recovery.

### Protocol development

Prior to this project, the BHCC’s strategy for buprenorphine initiation was to wait as close as possible to 72 hours from the last use of fentanyl and then start SL buprenorphine at 1 to 4 mg. Despite this cautious approach, some patients still experienced BPOW, and stabilization during transition to buprenorphine lasted for days. To address these challenges, the medical director of the BHCC (JE) instituted a KABI protocol as a clinical quality improvement intervention, with the aims of improving patient comfort and shortening length of stay (LOS). The idea for this protocol grew out of a presentation at the American Society of Addiction Medicine 54th Annual Conference [51] by two of the authors (LG and TH) and Andrew Herring, MD, with data later published as a pilot case series [42]. In that model, patients self-administered 16 mg SL ketamine compounded troches over several days for treatment of spontaneous fentanyl withdrawal and for both prevention and treatment of BPOW to facilitate at-home initiation of buprenorphine.

JE developed a high-dose buprenorphine initiation protocol with IM ketamine as premedication for prophylaxis against BPOW. The high-dose buprenorphine initiation strategy was chosen to achieve the dual goals of a high initiation completion rate and a short LOS. A SL buprenorphine dose of 8 mg (in the form of the combination buprenorphine/naloxone 8/2 mg tablet) was chosen based on the reported tolerability of 8–16 mg for the initial buprenorphine dose in an emergency department setting [46].

The IM administration route was selected due to simpler access and lower cost than SL ketamine troches, which would involve sending patient-specific prescriptions to a compounding pharmacy. The cost of a 10-vial box of 20 mL vials of ketamine 10 mg/mL–enough to treat 200 patients–was $88.82 through the facility’s medical supplies wholesaler. The cost was therefore about $0.44 per 10 mg injection. A 10 mg IM ketamine dose was chosen to achieve a stronger effect than the 16 mg SL ketamine doses in the pilot case series [42] while still limiting cognitive effects to a sub-dissociative level [52] to be safe in a setting without specialized medical resources. Ketamine’s SL bioavailability is approximately 25% (0.06 mg/kg for a 16 mg SL dose in a 70 kg person) while its IM bioavailability is approximately 93% (0.13 mg/kg for a 10 mg IM dose in a 70 kg person) [30]. A standard 10 mg dose was chosen instead of weight-based dosing to simplify the protocol and based on the success of fixed-dose sublingual administration in the pilot case series [42]. The fixed low dose was below the dissociative level regardless of weight in that case series.

### Participants

Participants eligible for inclusion in this report were adults presenting to the crisis center between June 1, 2023 and July 31, 2024 who were treated with the ketamine protocol. Inclusion criteria for the ketamine protocol were: 1) self-reported fentanyl use within the previous 24 hours, 2) a desire to initiate buprenorphine treatment to facilitate abstinence from fentanyl, and 3) at least moderate opioid withdrawal symptoms, defined as a Clinical Opiate Withdrawal Scale (COWS) score of 9 or greater (except patient #45, whose COWS score was 8). Exclusion criteria included inability to provide consent or contraindications to ketamine administration (e.g., active psychotic disorder or severe cardiovascular disease). Due to the lack of CLIA-waived point-of-care testing for fentanyl at the outset of this project, fentanyl exposure was determined by self-report in the initial cohort of 20 patients. Objective verification with urine toxicology was implemented in the second cohort of 30 patients when testing became available. Patients positive for and/or withdrawing from other substances were not excluded.

To compare LOS with the pre-protocol period, the quality improvement team identified 24 patients admitted with a diagnosis of OUD from May 2021 to May 2023 who had self-reported recent fentanyl use and successfully completed buprenorphine initiation. Successful initiation was defined as the patient having been transitioned to buprenorphine and discharged with a maintenance dose.

### Ethical considerations

Verbal consent was obtained from patients after explaining to them that 1) ketamine was being used off-label, 2) the potential risks of ketamine use including blood pressure elevation and dissociation, and that 3) ketamine had not been widely studied for this indication. De-identified data were maintained to determine the success of the quality improvement exercise. The University of South Alabama Institutional Review Board determined that this data analysis was exempt from IRB review as it did not meet the federal definition of human subjects research.

### Protocol

Patients were started on the protocol after at least 12 hours had elapsed since last fentanyl use. The previous practice at the BHCC had been to wait as close to 72 hours as possible after last fentanyl use before initiating buprenorphine. It was thought that waiting at least 12 hours from last use was a reasonable compromise to decrease the risk of BPOW but still allow patients to complete initiation within 24 hours of presentation to the BHCC. If protocol initiation occurred on the day of presentation, no comfort medications were given in advance. If patients presented at night at a time that a physician was not available, comfort medications including gabapentin, clonidine, loperamide, lorazepam, ondansetron, ibuprofen, and acetaminophen were offered until protocol initiation the next morning. The protocol was to administer IM ketamine 10 mg, and 30 minutes later to administer SL buprenorphine 8 mg. COWS score was assessed at the time of protocol initiation (the baseline), 30 minutes after ketamine, and 30 minutes after buprenorphine. All COWS assessments were performed by either of two of the authors, JE and ZO. Although formal inter-rater reliability was not tested, JE trained ZO in COWS assessment and ensured consistency in rating at the beginning of data collection. An increase in COWS ≥6 at 30 minutes after buprenorphine was classified as BPOW [50]. For continued COWS scores of 9 or higher, patients were treated symptomatically with additional doses of buprenorphine, ketamine, and/or other comfort medications based on physician judgement and patient preference. Patient treatment was conducted under continuous observation of medical and nursing staff who were present in the room. Vital signs were monitored every 4 hours, and there were no vital signs abnormalities requiring emergent treatment.

**Fig. 1:**
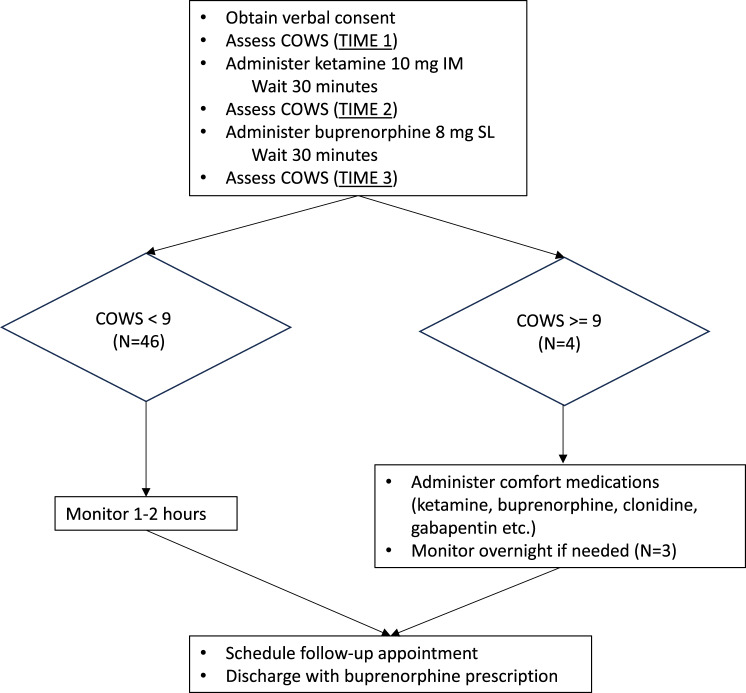
Procedures and outcomes

Beyond 30 minutes after buprenorphine, patients with a COWS score of 8 or lower were monitored for another one to two hours and discharged with a prescription for buprenorphine (typically buprenorphine/naloxone 8/2 mg twice daily) and a follow-up appointment within one week with an addiction medicine physician at the AltaPointe Health substance use disorders clinic (Fig. 1).

### Measures

Quality improvement measures collected were: 1) COWS scores at baseline, 30 minutes after ketamine, and 30 minutes after buprenorphine; 2) change in COWS score from baseline to 30 minutes after ketamine and to 30 minutes after buprenorphine; 3) percent of patients receiving additional medications and percent of patients requiring overnight stay; and 4) LOS from admission to discharge in hours. Additional measures performed for this analysis were prescription dispensed (by review of the state prescription monitoring program) and follow-up visit within 30 days (by review of the AltaPointe Health electronic medical record).

### Statistical analysis

A linear mixed-effects model was conducted to examine change in COWS scores over time, from baseline through the two follow-up measurements, controlling for covariates of age, sex, and fentanyl verification method, and accounting for within-subject correlation across timepoints that will be present due to the repeated measurements of each individual. The model included a random intercept to account for repeated measurements. We then ran post-hoc estimated marginal means and conducted pairwise comparisons between individual timepoints, with Cohen’s d calculated for each pairwise change.

Linear regression was conducted to examine relationships between treatment and LOS, controlling for covariates of age and sex. Because LOS was right skewed, we log transformed the variable before running analyses.

## Results

Over a thirteen-month period, 50 patients with recent fentanyl use agreed to buprenorphine initiation with the ketamine protocol. Their median age was 35.5 years (SD 7.3), 29 (58%) were male, 5 (10%) were Black, 1 (2%) was Hispanic, 1 (2%) was Native American, 43 (86%) were White, and 9 (18%) were unstably housed. No patients met exclusion criteria.

Full model results can be found in Table 1. Individual patients’ COWS scores at each time point and LOS are shown in Table 2.

**Table 1:** Mixed-effects model results

Variable	Coefficient	Std. Error	z	p-value	95% CI
Time 1: 30 min after ketamine	−7.34	0.53	−13.91	< 0.001	−8.37, −6.31
Time 2: 30 min after buprenorphine	−9.50	0.53	−18.00	< 0.001	−10.53, −8.47
Age (years)	−0.03	0.05	−0.55	0.580	−0.12, 0.07
Sex (Male = 0, Female = 1)	1.39	0.69	2.00	0.045	0.03, 2.75
Fentanyl Verification (Self-report = 1, Urine = 2)	−2.86	0.69	−4.15	< 0.001	−4.21, −1.51
Intercept	15.68	1.97	7.96	< 0.001	11.81, 19.54

**Table 2:** Demographics, LOS, and COWS scores

ID	Age	Sex	Race	Unstable Housing	LOS (hours)	COWS TIME 1	COWS TIME 2	COWS TIME 3	% Reduced at TIME 2 (SD)	% Reduced at TIME 3 (SD)
1	36	F	White		99	14	3	1	79%	93%
2	45	M	White		30	14	5	1	64%	93%
* 3	24	F	White		23	**13**	**4**	**6**	**69%**	**54%**
4	32	M	Black		5	13	9	8	31%	38%
5	48	M	White	X	3	13	12	6	8%	54%
6	54	M	White	X	4	13	1	1	92%	92%
* 7	41	F	White		6	**12**	**3**	**7**	**75%**	**42%**
8	52	M	White	X	5	13	9	1	31%	92%
9	40	M	White		50	16	4	1	75%	94%
10	38	F	White		2	16	7	6	56%	63%
11	35	M	White		98	9	8	2	11%	78%
* 12	32	M	White		47	**12**	**9**	**18**	**25%**	**-50%**
13	37	F	White		18	12	7	3	42%	75%
14	42	M	White	X	74	17	5	5	71%	71%
15	28	M	White		23	15	9	6	40%	60%
16	35	M	White	X	3	10	5	0	50%	100%
17	46	M	White		29	15	4	1	73%	93%
18	33	F	White		3	13	6	0	54%	100%
19	29	M	White		148	16	6	4	63%	75%
20	30	F	White		4	9	4	1	56%	89%
21	41	F	White		7	21	10	6	52%	71%
22	34	M	White	X	4	20	5	1	75%	95%
* 23	24	M	Black	X	22	**12**	**1**	**2**	**92%**	**83%**
24	33	M	White		23	14	8	7	43%	50%
25	44	F	White		3	14	10	5	29%	64%
26	43	M	White		3	14	6	1	57%	93%
27	42	M	White		2	15	15	13	0%	13%
28	33	F	White		28	14	0	0	100%	100%
29	30	F	Black		4	13	5	4	62%	69%
30	23	F	White		5	15	9	9	40%	40%
31	33	F	White		95	15	4	2	73%	87%
* 32	26	M	Hispanic		18	**21**	**4**	**10**	**81%**	**52%**
33	30	F	White		7	10	10	0	0%	100%
34	31	M	White		7	10	3	1	70%	90%
35	34	F	White		26	14	4	4	71%	71%
36	43	M	White	X	7	12	8	5	33%	58%
37	36	M	White		3	11	1	0	91%	100%
38	34	F	White		2	13	5	3	62%	77%
39	42	M	White		2	13	6	3	54%	77%
40	49	M	White		4	9	0	0	100%	100%
* 41	41	F	Black		49	**12**	**4**	**6**	**67%**	**50%**
42	29	F	Native American		28	13	4	1	69%	92%
43	44	M	White		26	21	17	16	19%	24%
44	32	M	White	X	3	12	9	8	25%	33%
45	44	M	White		52	8	3	3	63%	63%
46	36	F	White		46	13	7	3	46%	77%
47	38	F	Black		3	15	5	0	67%	100%
48	41	F	White		7	12	10	6	17%	50%
49	30	M	White		25	16	13	5	19%	69%
50	30	M	White		2	12	6	2	50%	83%
Summary Statistics	36.5				7.0	13.6	6.2	4.1	54% (26%)	71% (28%)

Table 1 key: TIME 1 = baseline, TIME 2 = 30 minutes after ketamine, TIME 3 = 30 minutes after buprenorphine. Asterisk ‘*’ next to the patient number and bold typeface indicates that the patient had a V-shaped COWS score trajectory. SD = standard deviation. Patients are numbered in sequence of admission at the facility. Note: Summary statistics are presented as mean (standard deviation) for Clinical Opiate Withdrawal Scale (COWS) score and Percentage Reduction. Age and Length of Stay (LOS) are presented as median to account for outliers

The overall linear mixed-effects model was significant, X^2^(5) = 376.39, *p* < 0.0001, suggesting a strong effect of change over the timepoints measured. Compared to baseline scores, COWS scores 30 minutes after ketamine (time 2; b = −7.34, *p* < 0.0001, 95% CI [−8.37, −6.31]) and 30 minutes after buprenorphine (time 3; b = −9.50, *p* < 0.001, 95% CI [−10.53, −8.47]) decreased significantly. Adjusted mean COWS scores were 13.6 (range 8–21, SD 2.9, 95% CI [12.62, 14.54]) at baseline; 6.2 (range 0–17, SD 3.6, 95% CI [5.28, 7.20]) 30 minutes after ketamine; and 4.1 (range 0–18, SD 4.0, 95% CI [3.12, 5.04]) 30 minutes after buprenorphine. At 30 minutes after ketamine, the average change from baseline in COWS score was −7.34 points (SD = 3.85), Cohen’s d was −1.91, a very large effect size. At this timepoint, 48/50 (96%) had a decrease in COWS score including nine patients with COWS score 0–3; two patients had no change in COWS score. At 30 minutes after buprenorphine, the average change from baseline in COWS score was −9.50 points (SD = 4.02), Cohen’s d was −2.36, an extremely large effect size. At this timepoint, 36/50 (72%) had a further decrease in COWS score including 27/50 (54%) with COWS score 0–3; 6/50 (12%) had an increase in COWS score compared to the value after ketamine, including 2/50 (4%) who met criteria for BPOW; and a total of 49/50 (98%) had a decrease in COWS score from baseline.

Age was not significantly related to COWS score (*p* = 0.58). Sex (b = 1.39, *p* = 0.045, 95% CI [0.03–2.75]) and fentanyl verification method (b = −2.86, *p* < 0.001, 95% CI [11.81–19.54]) were significantly related to the COWS scores in the model, suggesting that females had, on average, slightly higher COWS scores than males and that patients whose fentanyl use was verified by a point of care urine test, rather than by self-report, had COWS scores that were on average 2.9 points lower (i.e., objective verification was associated with less severe withdrawal scores).

An examination of random-effects variance estimates indicated moderate between-patient variability (variance = 3.20, 95% CI [1.58, 6.48]) and residual variance (variance = 6.96, 95% CI [5.28, 9.18]). A likelihood ratio test confirmed the choice of using a mixed model, as it fit significantly better than a standard linear model (X^2^ = 13.42, *p* = 0.0001).

Seven patients received additional medications during the first four hours: seven received additional buprenorphine (8 mg for six patients, 16 mg for one patient); four patients received additional ketamine (average 15 mg); and three received other comfort medications. Three of 50 patients (6%) stayed overnight due to a persistently elevated COWS score including the one patient whose COWS score after buprenorphine rose above its baseline value. Every other patient was stable enough for discharge within several hours after the first buprenorphine dose. No patient reported any adverse effect of ketamine.

Geometric mean LOS for patients receiving the ketamine protocol intervention was approximately 79% shorter than it was for patients who did not receive the intervention (i.e. the 24-patient pre-protocol comparison group)(exp(b) = 0.21). Neither the age nor sex covariate was significant (both *p* > 0.05). Because the LOS data was skewed, median LOS is most appropriate (as opposed to mean LOS) to consider descriptively. The median LOS was 7.0 hours (range 2–148) among patients receiving the intervention, and 66.0 hours (range 20–135) among patients who did not receive the intervention. The quality improvement protocol was therefore associated with a decrease in median LOS of 89%.

The multiple linear regression conducted to examine relationships between intervention status and log-transformed LOS, controlling for sex and age, was statistically significant, F(3,70) = 11.26, *p* < 0.001, and explained approximately 32.6% of the variance in the outcome (R^2^ = 0.326). Adjusting for covariates, intervention status was significantly related to LOS (b = −1.57, SE = 0.28, *t* = −5.65, *p* < 0.001. This indicates that patients who received the ketamine intervention had a significantly shorter LOS than those who did not.

Within 30 days after the initial buprenorphine dose, all 50 patients (100%) were dispensed at least one prescription for buprenorphine from a pharmacy, and 26/50 patients (52%) attended a follow-up visit with a physician.

No protocol deviations or incomplete collection of COWS score data occurred. Follow-up was assessed on all 50 patients.

## Discussion

This report describes a novel buprenorphine initiation protocol for fentanyl-dependent patients that uses an initial dose of 8 mg of SL buprenorphine following premedication with IM ketamine at a low, sub-dissociative dose (10 mg = ~0.13 mg/kg). The reductions in COWS score, both after ketamine premedication and again after buprenorphine, were statistically significant. Half of patients had nearly complete resolution of withdrawal symptoms (COWS 0–3) by 30 minutes after buprenorphine. While two patients met criteria for BPOW, only one had an increase in COWS above the baseline level. Almost all patients were stable enough for discharge from the crisis center within hours after the first buprenorphine dose.

Previously described high-dose buprenorphine initiation strategies reliably allowed patients to reach a therapeutic dose quickly [16, 46–48]. The unique characteristic of this ketamine-assisted buprenorphine initiation protocol was the rapid reduction in withdrawal symptoms in most patients even before the initial buprenorphine dose.

**Fig. 2:**
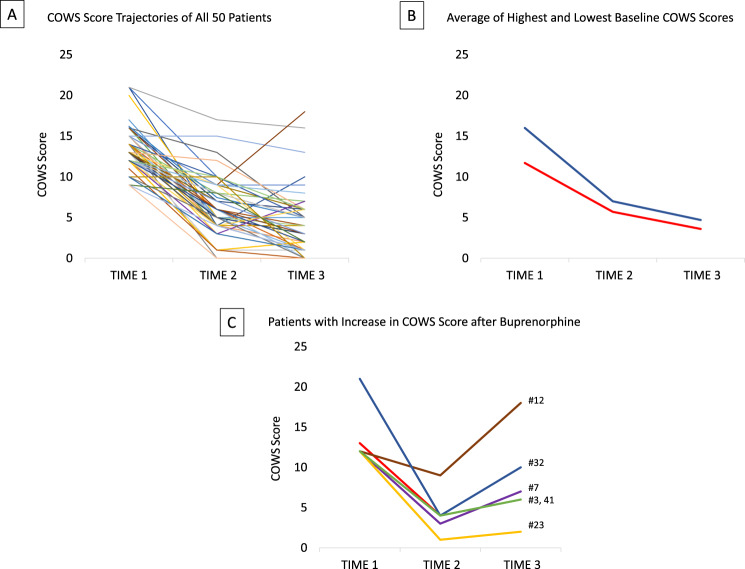
Trajectories of COWS scores. **A**) COWS score for all patients at baseline (TIME 1), 30 minutes after ketamine (TIME 2), and 30 minutes after buprenorphine (TIME 3). **B**) Average COWS score at baseline (TIME 1), 30 minutes after ketamine (TIME 2), and 30 minutes after buprenorphine (TIME 3). Blue line: COWS scores 14–21 (*N* = 22). Red line: COWS scores 8–13 (*N* = 28). COWS score for most patients got progressively lower after ketamine and then buprenorphine. **C**) Patients with a V-shaped trajectory, where COWS score dropped from the baseline value after ketamine (from TIME 1 to TIME 2) and then rose after buprenorphine (from TIME 2 to TIME 3). In all cases except #12, the COWS score was lower at TIME 3 than TIME 1. One patient’s trajectory is barely visible as the points overlap those of another patient

Figure 2A shows the trajectory of COWS scores for all 50 patients. As shown in Fig. 2B, the average and by far most common trajectory of COWS scores was a decrease after ketamine and a further decrease after buprenorphine, similar for patients with severe (blue line) and moderate (red line) baseline withdrawal. As shown in Fig. 2C, while only one patient had a higher COWS score after buprenorphine compared to baseline (an increase from 12 to 18 for patient #12), five others (patients #3, #7, #23, #32 and #41) had higher COWS score after buprenorphine compared to the value after ketamine. These five patients are among those with the steepest average drop from baseline after ketamine: 77% vs. 54% for all patients. The distinct ‘V-shaped’ trajectory, characterized by robust initial relief from ketamine followed by a transient return of symptoms upon buprenorphine administration, is likely associated with multiple unknown individual factors that likely contributed, such as the patients’ prior opioid exposure intensity and duration, the specific opioid and the interval since last exposure, as well as non-opioid factors such as anxiety, other medications administered, and substances ingested.

Ketamine reduced fentanyl withdrawal in all but two patients (#27 and #33) whose COWS scores (15 and 10) did not drop until 30 minutes after buprenorphine. Ketamine’s reversal of fentanyl withdrawal in most patients is consistent with results in the previous report of KABI [35]. In that study, repeated doses of ketamine could sustain patient comfort over several days before the first dose of buprenorphine.

The results reported here indicate that the discomfort associated with a high COWS score was not required to avoid BPOW. Figure 2B shows that the COWS score dropped after ketamine and dropped further after buprenorphine in both high (14–21) and moderate (8–13) baseline COWS score groups. It is possible that ketamine premedication could reliably reduce the risk of BPOW even in patients with mild or no withdrawal symptoms, as long as sufficient time has elapsed since last fentanyl use to leave enough mu-opioid receptors unoccupied. Repeated doses of ketamine may be an effective way to allow time to pass while avoiding withdrawal symptoms [42].

A shorter inpatient LOS provides health system efficiencies and lower cost [53]. The median LOS was much shorter during the protocol period than the pre-protocol period (7.0 vs. 66.0 hours). The median values in both groups were somewhat inflated above the actual time needed for symptom management: each patient’s LOS included medical and administrative time before and after buprenorphine initiation, and the median value was further elevated by the delayed discharge of some patients with disposition issues such as unstable housing, no available transportation, or referral to a residential rehabilitation program.

The authors believe that the most important contributor to the protocol’s shorter LOS was the use of the rapid high-dose buprenorphine initiation strategy. In the pre-protocol comparison group, a multi-day low-dose buprenorphine taper was commonly used. It was also more common in the pre-protocol period to use buprenorphine at low doses for several days to manage acute withdrawal symptoms with the intent to start naltrexone, an option deemed reasonable at that time due to the relative rarity of fentanyl in the local drug supply. The high-dose strategy quickly became acceptable to patients after the BHCC’s positive early experience with ketamine premedication. The quality improvement team is not aware of any qualified patients who declined the protocol.

The comparison group was smaller than the protocol group because fentanyl was just entering the community drug supply in Mobile between 2021 and 2023, and fewer patients self-identified as fentanyl-dependent.

The 100% pharmacy dispense rate, while high, was comparable to that of other studies on buprenorphine prescriptions: 97.9% after audio-only telehealth buprenorphine initiation [54], and 91% after a bridge clinic telemedicine visit [55]. Likely contributors to the high rate of prescription dispensing are the presence of an AltaPointe Health pharmacy across the street from the BHCC, and an Alabama state grant that pays for buprenorphine for uninsured patients who enroll in their outpatient substance use disorders clinic.

The 52% follow-up rate is within the range of some other studies of buprenorphine initiation: 50.6% [47] and 86% [46] after an initial prescription at an emergency department. Patients in urgent and emergency settings such as the BHCC and emergency departments tend to be seeking immediate relief rather than ongoing treatment. In any case, this report assesses only the effectiveness of the buprenorphine initiation protocol. Many factors would influence follow-up rate independent of the initiation protocol, such as psychiatric, social, and transportation issues.

For clinicians considering implementing the protocol described here, use of the lowest concentration of ketamine (10 mg/mL) and a small syringe size (ideally a 1 mL insulin syringe) would provide the greatest safety. A higher concentration (20 mg/mL or 100 mg/mL) and larger syringe size would introduce risk of inadvertent administration of a dissociative or anesthetic-level dose of ketamine by miscalculating the volume to be injected. While respiratory depression does not occur with ketamine even at anesthetic-level dosing of 1–2 mg/kg (70–140 mg in a 70 kg person) [23, 24], an unexpected altered state of consciousness would pose medical, psychological, and behavioral risks as discussed in the Introduction.

This report’s protocol that used sub-dissociative dose IM ketamine as premedication for a high-dose buprenorphine initiation could be adapted for use in other settings where buprenorphine treatment is offered, including inpatient, emergency department, residential treatment, withdrawal management, crisis center, outpatient, carceral, or street medicine and warrants further study in these settings. Importantly, ketamine is a Schedule III controlled substance. In outpatient settings and street medicine, IM administration by a health care provider may be safer than oral or sublingual self-administration as it avoids the risk of misuse among patients with a substance use disorder in which impaired self-control is an integral part of the disease.

If the favorable results observed here are confirmed in other studies, sub-dissociative dose ketamine could be incorporated into the range of tools available, helping to address the significant apprehensiveness among both potential patients and providers that prevents many people with a fentanyl use disorder from initiating treatment with buprenorphine.

## Limitations

The limitations of this report include its observational nature at a single site in a naturalistic clinical setting. We do not have a comparison group on COWS scores and completion rates from the same time period since all patients offered ketamine consented to receive it. Data available from the pre-protocol period is limited due to lack of systematic data collection on COWS scores and completion rates during that time.

There are limitations due to the nature of this study as a quality improvement protocol rather than prospective research. The purpose of this quality improvement protocol was to explore whether adjunctive low-dose ketamine could improve reliability of buprenorphine initiation and patient comfort during the process. While we did not have a control group, the magnitude and rapidity of COWS score reduction observed in this cohort was substantially greater than what we typically see with buprenorphine alone in our clinical experience. These findings are preliminary and hypothesis-generating, warranting confirmation in a randomized controlled trial.

We did not collect blood pressure at standardized time points, so we were unable to assess the effect of ketamine on blood pressure. When designing the protocol, we anticipated that ketamine would be protective against the cardiovascular stress of precipitated withdrawal, based on vital signs measured in a previous prospective study [38] and the reduction in withdrawal symptoms among patients who used ketamine in advance of each buprenorphine dose [42]. Future prospective studies could assess the cardiovascular effect of ketamine by measuring pre-ketamine and post-ketamine blood pressure and heart rate in patients undergoing buprenorphine initiation.

Sex and fentanyl verification method were associated with baseline COWS scores, suggesting that these factors be taken into consideration in future studies and clinical applications. Participants and clinicians were non-blinded to medications given. COWS scoring was not recorded beyond 30 minutes after buprenorphine, although supplemental comfort medications were provided to 7 patients for withdrawal symptoms beyond 30 minutes. Fentanyl use by patient report for the first 40% of patients was less reliable than the urine testing available for the last 60%, though there was no obvious difference in outcome between the early and late patients. The COWS score measured by clinicians is somewhat imprecise and may not accurately reflect the subjective patient experience. Some patients may have experienced dissociative effects of ketamine that they did not report. The follow-up clinic visit rate did not include any patients who established care at clinics outside the AltaPointe Health system. Additionally, patients in the two LOS cohorts were not randomly assigned or matched, but rather the comparison cohort was a convenience sample taken from the time period before the intervention began.

A contributing factor to the higher LOS in the comparison group may have been exposure to tianeptine, a non-FDA-approved antidepressant that was previously highly prevalent on the Alabama Gulf Coast, typically obtained legally in gas stations and convenience stores. That drug’s complex pharmacology can complicate the withdrawal process in part due to agonist action at the mu-opioid receptor [56]. However, the tianeptine exposure rate in Alabama rapidly declined after it was classified as a controlled substance on March 15, 2021, two months before the start of the pre-protocol comparison group period [57]. The comparison group was drawn only from patients who reported fentanyl use. Although it is possible that some of those patients may have had exposure to tianeptine as the facility did not test for tianeptine, no one in the comparison group reported tianeptine use in addition to fentanyl.

## Conclusions

In this observational study in a high-acuity crisis residential setting, sub-dissociative dose IM ketamine was a well-tolerated and inexpensive premedication for a high-dose buprenorphine initiation in a protocol that improved outcomes compared to prior practice. Nearly all patients undergoing this simple protocol had rapid reduction of spontaneous fentanyl withdrawal symptoms, half of patients had nearly complete resolution of withdrawal symptoms within one hour, and LOS was lower than with strategies used previously at the facility.

The protocol presented here has the characteristics needed to gain wide acceptance: it is simple to explain and implement and uses inexpensive and easily accessible medication. Widespread use of this protocol could increase uptake of buprenorphine among fentanyl-dependent patients and reduce overdose deaths. Formal research is warranted.

## Data Availability

All data generated or analyzed during this study are included in this published article.

## Abbreviations

BHCC: AltaPointe Health Behavioral Health Crisis Center
BPOW: Buprenorphine-precipitated opioid withdrawal
COWS: Clinical Opiate Withdrawal Scale
FDA: Food and Drug Administration
IM: Intramuscular
KABI: Ketamine-assisted buprenorphine initiation
LAI: Long-acting injectable
LOS: Length of stay
NMDAr: N-methyl-D-aspartate-type glutamate receptor
OUD: Opioid use disorder
SL: Sublingual
